# Acute Presentation of Asymptomatic Giant Endometrioma in Pregnancy: A Case Report

**DOI:** 10.7759/cureus.49580

**Published:** 2023-11-28

**Authors:** Pooja Sahu, Deepthy Balakrishnan, Bhavya Singh

**Affiliations:** 1 Obstetrics and Gynaecology, All India Institute of Medical Sciences, Bhubaneswar, Bhubaneswar, IND

**Keywords:** huge ovarian tumor, maternal and fetal outcome, pregnancy, asymptomatic, endometrioma

## Abstract

Endometriomas are associated with severe endometriosis and are uncommon in asymptomatic women. Reported cases of giant endometriomas are few especially in pregnancy. Decidualization of endometriomas can mimic malignancies in pregnancy. Fetal outcomes can be good after excision of large endometriomas in the 2nd trimester.

We present a case of giant endometrioma diagnosed in an asymptomatic woman who developed symptoms after becoming pregnant. Clinical findings, investigations, and histopathology were consistent with ovarian endometrioma. Maternal and fetal outcomes were good after the excision of the mass.

## Introduction

The reported incidence of endometriosis in asymptomatic women varies between 2%-50% [1]. There is a lack of data on the incidence of endometrioma in asymptomatic women. Endometrioma is commonly associated with severe endometriosis [1]. As the severity of symptoms does not correlate with the stage of disease, endometriomas can be asymptomatic. Ovarian endometriomas are typically 10-15 cm in diameter [2] and there is a scarcity of published data on giant endometriomas in pregnancy. We report a case of huge endometrioma of 30 cm in its largest diameter in a pregnant woman who presented with symptoms of short duration in the second trimester.

## Case presentation

Case history

**Figure 1:**
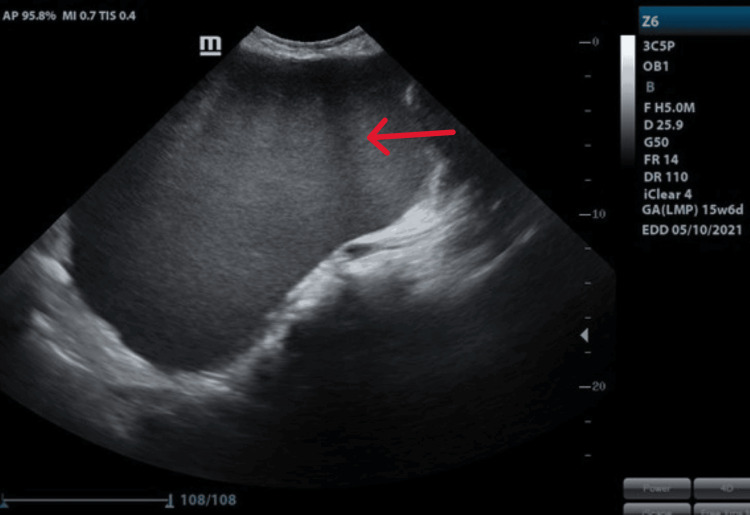
Ultrasound finding of ovarian endometrioma

A 30-year-old primigravida at 15 weeks 4 days presented with pain in the abdomen and gradual abdominal distension for the past 3 months. This was a spontaneous conception with no prior history of dysmenorrhoea, dyspareunia, or pelvic pain. Her past menstrual cycles were regular. Examination revealed a 36-week-sized firm mass with a smooth surface and restricted mobility. In ultrasonography, a huge unilocular cyst measuring approximately 30 x 20 cm with a thick wall was visualized showing a typical ground glass appearance (Figure 1). There was no increased vascularity in Doppler. Serum CA 125 was normal.

Treatment

**Figure 2:**
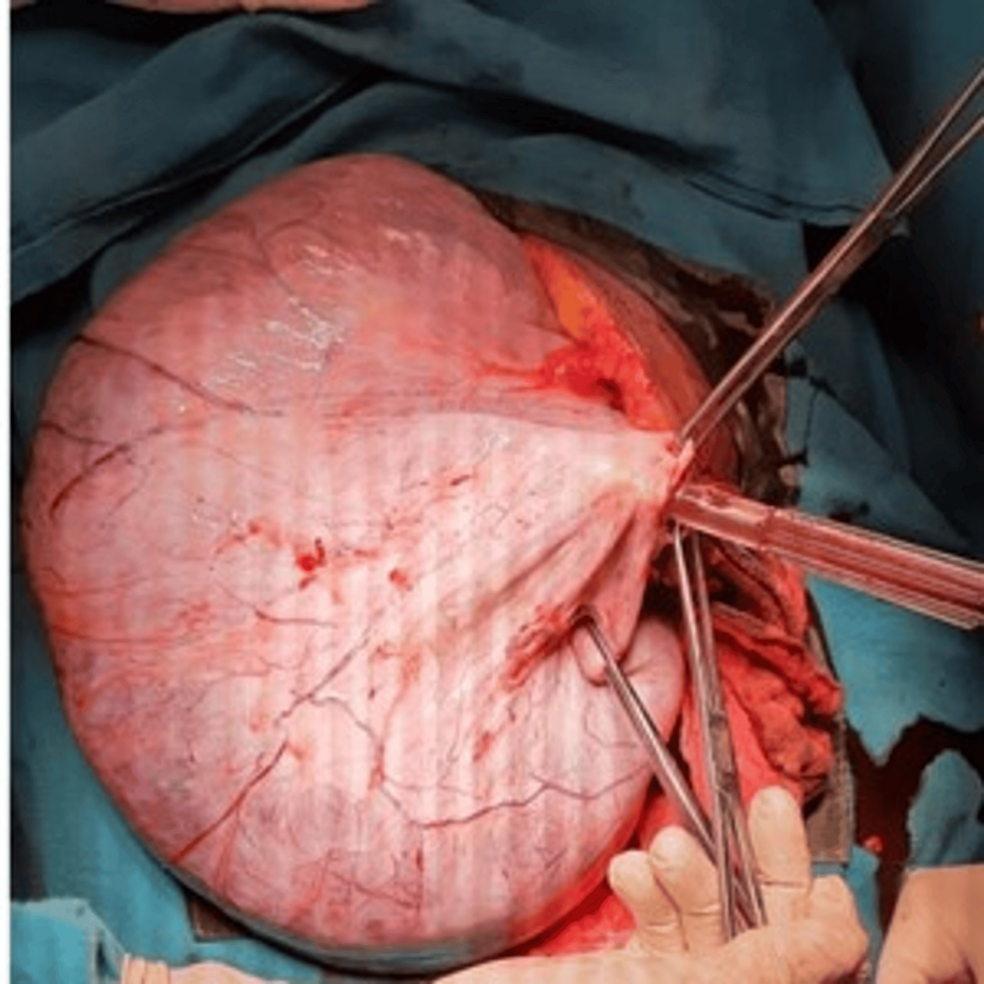
Intraoperative finding of a huge ovarian mass

An exploratory laparotomy with a midline vertical incision was performed. A huge cystic greyish-white mass with a thick wall and smooth surface filling the entire abdomen was seen intraoperatively arising from the left ovary (Figure 2). There was no evidence of endometriotic deposits elsewhere in the abdomen or pelvis. The cyst was punctured and around 8150 ml of chocolate-colored fluid was suctioned out. The uterus was 16 weeks in size corresponding to the period of gestation, and the right ovary and tube were normal in appearance. Due to the huge size of the cyst, a left salpingo-oophorectomy was performed. Fetal cardiac activity was checked and was found to be normal postoperatively. The postoperative period was uneventful.

Pathological investigations

**Figure 3:**
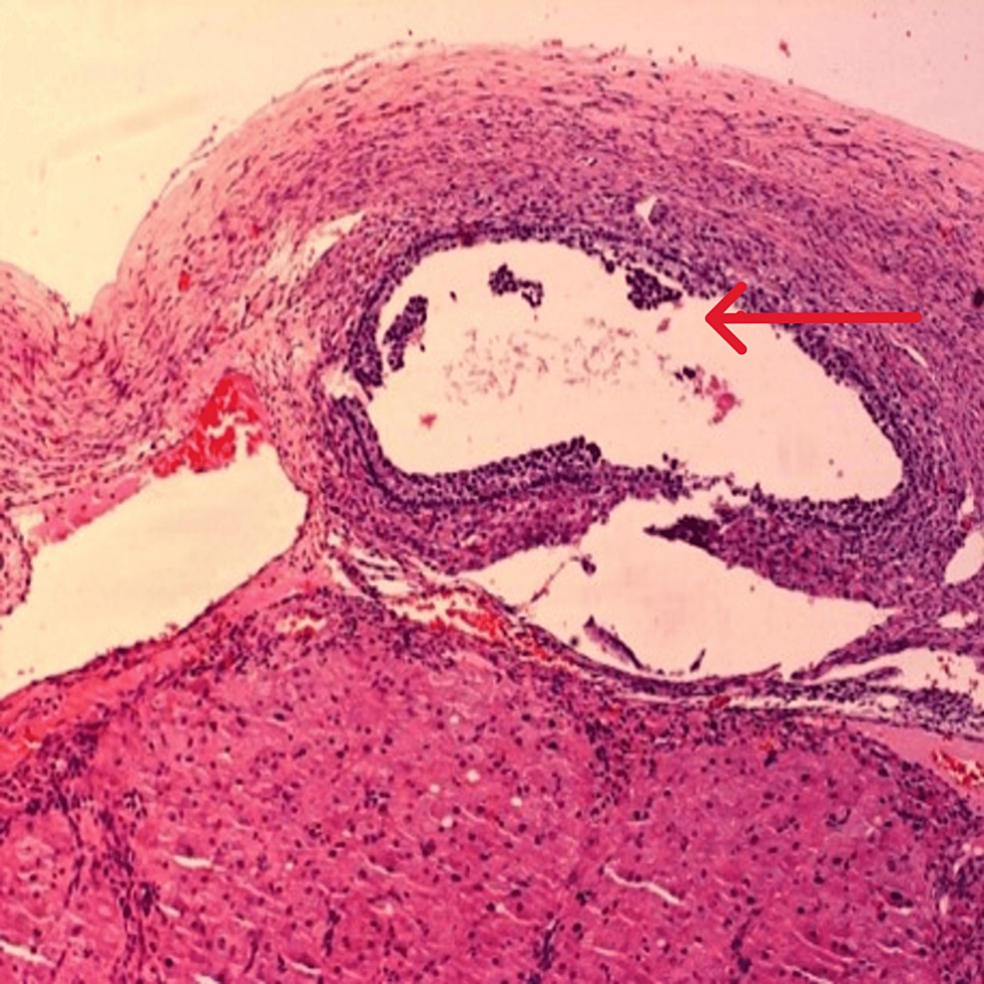
Microscopy showing features of endometrioma

**Figure 4:**
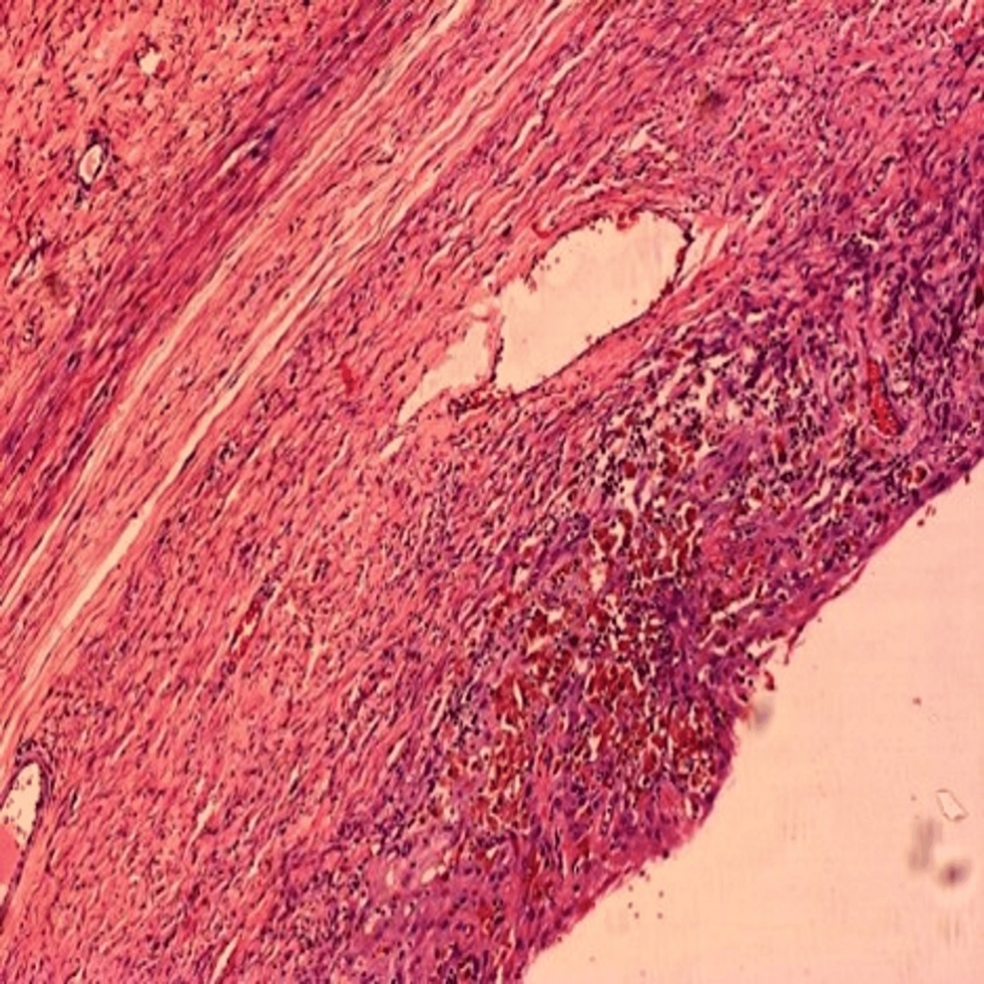
Microscopy showing features of endometrioma

On gross pathological examination, the cyst was 30 x 20 cm with a thick wall ranging in size from 0.2 cm to 0.3 cm. The outer surface was greyish-white to brown in color and showed prominent blood vessels. The inner surface had large areas of hemorrhage and thickening. Microscopy revealed a benign cyst with decidualized stroma containing hemosiderin-laden macrophages and lymphocytic infiltrates consistent with our diagnosis of endometrioma (Figure 3, 4).

Outcome

The patient was followed up antenatally. She had no complications in the antenatal period and had a full-term delivery of a male baby weighing 2895 gm by lower segment Caesarean section (LSCS)due to fetal distress at 40 weeks. Both mother and baby were healthy and discharged on day 4 postoperatively.

## Discussion

There is less data on asymptomatic endometrioma [1]. It is uncommon to have a huge endometrioma in an asymptomatic woman [3] and the absence of symptoms might have been due to the absence of deposits elsewhere in the pelvis. The reported prevalence of endometriomas in pregnancy is between 0.2% and 0.3% [4]. Endometriomas are seen in severe endometriosis [1], and the absence of deposits with a huge endometrioma is uncommon. This may be due to high progesterone levels in pregnancy which leads to apoptosis and regression of endometriosis. Literature review showed two reported cases of huge ovarian endometrioma in nonpregnant women. Ishikawa et al (1997) reported an endometrioma of 25 × 18 × 12 cm with 2500 ml of chocolate-colored fluid [5] and Yasar et al (2010) reported an endometrioma of 26 × 18 × 17 cm with 3250 ml of chocolate-brown fluid. [6] The endometrioma, in our case, was 30 × 20 cm with 8150 ml of chocolate-colored fluid and may be the largest endometrioma reported. Large endometriomas are rare in pregnancy and our case may be the largest ovarian endometrioma in pregnancy reported in the literature. MRI was not done as an ultrasound abdomen showed typical features of endometriosis with no features of decidualization. Tumor markers also were normal. Decidualization is seen only in 12% of cases [4]. Pregnancy outcomes were good in women operated for benign ovarian conditions, especially before 23 weeks of gestation [7], which was the same in our patient. A thick fibrous capsule containing a cluster of hemosiderin-laden macrophages due to repeated hemorrhage, which is the most specific pathologic feature of endometrioma, was seen on microscopy [8] confirming the diagnosis of ovarian endometrioma.

## Conclusions

Giant ovarian endometrioma is a rare, especially in pregnancy. Endometrioma may be a differential diagnosis of large ovarian masses in asymptomatic women. The diagnosis was established by clinical examination and ultrasonography and was confirmed by histopathology. The decision to perform surgery must be made with caution without delaying treatment in the event of a strong suspicion of malignancy and/or a complication.
